# Supplementary material for: A fault-intrusion-tolerant system and deadline-aware algorithm for scheduling scientific workflow in the cloud
Source: PeerJ Comput Sci. 2021 Nov 2;7:e747. doi: 10.7717/peerj-cs.747 (PMC8576554; doi:10.7717/peerj-cs.747)
Supplement: Supplemental Information 6 [file peerj-cs-07-747-s006.zip › Myproject1.1/docs/org/workflowsim/utils/OverheadParameters.html]

OverheadParameters


JavaScript is disabled on your browser.


- Overview
- Package
- Class
- Use
- Tree
- Deprecated
- Index
- Help

- Prev Class
- Next Class

- Frames
- No Frames

- All Classes

- Summary:
- Nested |
- Field |
- Constr |
- Method

- Detail:
- Field |
- Constr |
- Method


org.workflowsim.utils

## Class OverheadParameters

- java.lang.Object
- - org.workflowsim.utils.OverheadParameters

- ---

    

  ```
  public class OverheadParameters
  extends java.lang.Object
  ```

  This class includes all parameters that involve overheads

  Since:
  :   WorkflowSim Toolkit 1.0

- - ### Constructor Summary

    Constructors

    Constructor and Description

    `OverheadParameters(int wed_interval,
    java.util.Map<java.lang.Integer,java.lang.Double> wed_delay,
    java.util.Map<java.lang.Integer,java.lang.Double> queue_delay,
    java.util.Map<java.lang.Integer,java.lang.Double> post_delay,
    java.util.Map<java.lang.Integer,java.lang.Double> cluster_delay,
    double bandwidth)`

    Created a new OverheadParameters object.
  - ### Method Summary

    Methods

    | Modifier and Type | Method and Description |
    | --- | --- |
    | `double` | `getBandwidth()` Gets the bandwidth |
    | `java.util.Map<java.lang.Integer,java.lang.Double>` | `getClustDelay()` Gets the clustering delay |
    | `double` | `getClustDelay(Cloudlet cl)` Gets the clustering delay for a particular job based on the depth(level) |
    | `java.util.Map<java.lang.Integer,java.lang.Double>` | `getPostDelay()` Gets the postscript delay |
    | `double` | `getPostDelay(Job job)` Gets the postscript delay for a particular job based on the depth(level) |
    | `java.util.Map<java.lang.Integer,java.lang.Double>` | `getQueueDelay()` Gets the queue delay |
    | `double` | `getQueueDelay(Cloudlet cl)` Gets the queue delay for a particular job based on the depth(level) |
    | `java.util.Map<java.lang.Integer,java.lang.Double>` | `getWEDDelay()` Gets the workflow engine delay |
    | `double` | `getWEDDelay(java.util.List list)` Gets the workflow engine delay for a particular job based on the depth(level) |
    | `int` | `getWEDInterval()` Gets the interval |

    - ### Methods inherited from class java.lang.Object

      `clone, equals, finalize, getClass, hashCode, notify, notifyAll, toString, wait, wait, wait`

- - ### Constructor Detail


    - #### OverheadParameters

      ```
      public OverheadParameters(int wed_interval,
                        java.util.Map<java.lang.Integer,java.lang.Double> wed_delay,
                        java.util.Map<java.lang.Integer,java.lang.Double> queue_delay,
                        java.util.Map<java.lang.Integer,java.lang.Double> post_delay,
                        java.util.Map<java.lang.Integer,java.lang.Double> cluster_delay,
                        double bandwidth)
      ```

      Created a new OverheadParameters object.

      Parameters:
      :   `wed_interval,` - the interval of workflow engine
      :   `wed_delay,` - the list of workflow engine delay
      :   `queue_delay,` - the list of queue delay
      :   `post_delay,` - the list of postscript delay
      :   `cluster_delay,` - the list of clustering delay
      :   `bandwidth,` - the bandwidth
  - ### Method Detail


    - #### getBandwidth

      ```
      public double getBandwidth()
      ```

      Gets the bandwidth

      Returns:
      :   the bandwidth


    - #### getWEDInterval

      ```
      public int getWEDInterval()
      ```

      Gets the interval

      Returns:
      :   the interval


    - #### getQueueDelay

      ```
      public java.util.Map<java.lang.Integer,java.lang.Double> getQueueDelay()
      ```

      Gets the queue delay

      Returns:
      :   the queue delay


    - #### getPostDelay

      ```
      public java.util.Map<java.lang.Integer,java.lang.Double> getPostDelay()
      ```

      Gets the postscript delay

      Returns:
      :   the postscript delay


    - #### getWEDDelay

      ```
      public java.util.Map<java.lang.Integer,java.lang.Double> getWEDDelay()
      ```

      Gets the workflow engine delay

      Returns:
      :   the workflow engine delay


    - #### getClustDelay

      ```
      public java.util.Map<java.lang.Integer,java.lang.Double> getClustDelay()
      ```

      Gets the clustering delay

      Returns:
      :   the clustering delay


    - #### getClustDelay

      ```
      public double getClustDelay(Cloudlet cl)
      ```

      Gets the clustering delay for a particular job based on the depth(level)

      Parameters:
      :   `cl,` - the job

      Returns:
      :   the clustering delay


    - #### getQueueDelay

      ```
      public double getQueueDelay(Cloudlet cl)
      ```

      Gets the queue delay for a particular job based on the depth(level)

      Parameters:
      :   `cl,` - the job

      Returns:
      :   the queue delay


    - #### getPostDelay

      ```
      public double getPostDelay(Job job)
      ```

      Gets the postscript delay for a particular job based on the depth(level)

      Parameters:
      :   `cl,` - the job

      Returns:
      :   the postscript delay


    - #### getWEDDelay

      ```
      public double getWEDDelay(java.util.List list)
      ```

      Gets the workflow engine delay for a particular job based on the
      depth(level)

      Parameters:
      :   `cl,` - the job

      Returns:
      :   the workflow engine delay


- Overview
- Package
- Class
- Use
- Tree
- Deprecated
- Index
- Help

- Prev Class
- Next Class

- Frames
- No Frames

- All Classes

- Summary:
- Nested |
- Field |
- Constr |
- Method

- Detail:
- Field |
- Constr |
- Method
